# A Selective Medium for Screening Ceftazidime/Avibactam Resistance in Carbapenem-Resistant *Enterobacterales*

**DOI:** 10.3389/fmicb.2022.956044

**Published:** 2022-07-12

**Authors:** Weiliang Zeng, Wenli Liao, Yajie Zhao, Lingbo Wang, Hongyun Shu, Huaiyu Jia, Tao Chen, Ying Zhang, Tieli Zhou, Qing Wu

**Affiliations:** ^1^Department of Clinical Laboratory, Key Laboratory of Clinical Laboratory Diagnosis and Translational Research of Zhejiang Province, The First Affiliated Hospital of Wenzhou Medical University, Wenzhou, China; ^2^Department of Laboratory, Yongzhou Central Hospital, Yongzhou, China; ^3^Department of Medical Lab Science, School of Laboratory Medicine and Life Science, Wenzhou Medical University, Wenzhou, China

**Keywords:** ceftazidime, avibactam, medium, CRE, resistance, screening

## Abstract

Ceftazidime/avibactam (CZA) is an alternative antibiotic used for the treatment of infections caused by carbapenem-resistant *Enterobacterales* (CRE). However, the CZA-resistant CRE strains have been detected worldwide. Therefore, it is critical to screen CZA-resistant CRE strains in colonized patients or a specific population so as to rapidly implement infection control measures to limit their transmission. In this study, we developed a *Salmonella*-*Shigella* (SS) CZA-selective medium and assessed its performance to screen for clinical CZA-resistant CRE isolates in both pure-strain specimens and stool samples. A total of 150 non-duplicated isolates, including 75 CZA-susceptible and 75 CZA-resistant CRE pathogens, were tested by using the broth microdilution method and the SS CZA medium, respectively. The bacterial suspensions were serially diluted in the SS CZA medium, which showed excellent screening performance in both pure CZA-resistant CRE strain and the stool samples with the lowest detection limit of 10^1^-10^2^ and 10^1^-10^3^ CFU/ml, respectively. Notably, none of the susceptible isolates showed growth even at the highest dilution concentration of 10^8^ CFU/ml. Most importantly, the SS CZA medium demonstrated excellent performance in screening simulated clinical polymicrobial specimens. Moreover, its screening performance was unaffected by the different resistance determinants for tested isolates. Cumulatively, our data suggest that the SS CZA medium can be used as a promising selective medium to screen CZA-resistant CRE, irrespective of their resistance mechanisms.

## Introduction

Antibiotic resistance has become a major public health concern. Multidrug-resistant (MDR) pathogens are increasingly restricting the success of antibiotic treatments. In the past decades, carbapenems (such as imipenem, meropenem, and ertapenem) were demonstrated to possess a broad-spectrum antibacterial activity. In fact, they were considered the last resort for the treatment of infections caused by MDR *Enterobacterales* (including *Enterobacter cloacae, Escherichia coli*, and *Klebsiella pneumoniae*). However, presently, carbapenem-resistant *Enterobacterales* (CRE) have been detected globally. CRE is resistant to several antibiotics and is associated with a mortality rate of up to 50% (Ackley et al., 2020). In addition, a prospective cohort study revealed that 57% of patients were colonized with CRE (van Duin et al., 2020). Therefore, there is an urgent need to develop novel antibiotics to combat CRE infections.

Ceftazidime/avibactam (CZA) is a novel synthetic β-lactamase inhibitor combination that consists of the third-generation cephalosporin ceftazidime (CAZ) and a newly developed β-lactamase inhibitor, avibactam (AVI). CZA is an effective antimicrobial agent against several enzyme-producing microorganisms, including extended-spectrum β-lactamases (ESBLs)-producing *Enterobacterales* (Zhang et al., 2018). Increasing evidence supports that CZA can be used to treat bacterial infections caused by MDR Gram-negative bacteria, including CRE infections (Shields et al., 2016; Caston et al., 2017; Krapp et al., 2017; Tumbarello et al., 2019). Unfortunately, resistance to CZA among CRE has gradually increased across the world with the extensive use of CZA in clinics (Koren et al., 2017; Shields et al., 2017). It has been widely reported that mutations in class A β-lactamase contribute to the development of increased CZA resistance. In *E. coli* and *K. pneumoniae*, the mutations at different sites of KPC increased the minimum inhibitory concentration (MIC) of CZA to various degrees (Haidar et al., 2017; Hemarajata and Humphries, 2019). Some mutations in *bla*_CTX−M_ (Livermore et al., 2018) and *bla*_SHV_ (Winkler et al., 2015) genes have also been reported to contribute to increased resistance to CZA. In addition, mutations in class C β-lactamase AmpC have been reported in several studies. Strains with AmpC mutations have been reported in *Enterobacterales* (Livermore et al., 2018). The base-pair substitutions of class D β-lactamase *bla*_OXA−48_ may induce resistance to CZA (Frohlich et al., 2019). The early detection of potentially CZA-resistant strains is extremely important to prevent further bacterial infections and transmission.

According to the guidelines of the Clinical and Laboratory Standards Institute (CLSI) (Prater et al., 2019), the broth microdilution (BMD) method was used as the reference method to detect the susceptibility of *Enterobacterales* to CZA. However, it is difficult to conduct this method in general clinical laboratories considering the associated complication and time investment. Therefore, effective screening mediums are essential for early, quick, and accurate detection of CZA-resistant CRE isolates. Recently, a CZA-resistance screening medium, called SuperCAZ/AVI, was developed by Sadek et al. to detect CZA resistance among *Enterobacterales* and *Pseudomonas aeruginosa* based on the CHROMagar™ Orientation medium (Sadek et al., 2020). According to their findings, the SuperCAZ/AVI medium contains CAZ, AVI, daptomycin, and amphotericin B, and is a selective medium with both 100% sensitivity and specificity when the lower limit of detection was greater than the cutoff value of 10^3^ CFU/ml.

To design a screening medium appropriate for rectal swab specimens, it is important to select a selective medium that inherently inhibits contamination by Gram-positive bacteria and fungi, such as *Enterococcus* and *Candida*. The *Salmonella–Shigella* (SS) agar medium is a commonly used strong selective medium for the isolation and detection of intestinal pathogens in most clinical microbiology laboratories, especially for the isolation and culture of *Salmonella* and *Shigella* strains. However, it is concerning that certain component of the SS agar medium, such as sodium citrate and bile salts, can prevent the growth of *Candida* and Gram-positive bacteria such as *Staphylococcus* and *Enterococcus* strains. In fact, with the popularity of CRE, carbapenem-drug discs are adhered to the SS agar medium to screen for CRE strains in some hospital laboratories (Aleem et al., 2021). In this study, we developed an SS CZA medium based on the SS agar medium and the SuperCAZ/AVI medium, followed by the assessment of its performance to screen for clinical CZA-resistant CRE strains.

## Materials and Methods

### Bacterial Isolates

The tested strains were collected from the First Affiliated Hospital of the Wenzhou Medical University. The First Affiliated Hospital of Wenzhou Medical University of institutional ethics committee did not require the study to be reviewed or approved by an ethics committee considering its observational nature with the primary focus on bacteria and the no interventions made to patients.

A total of 150 non-duplicate CRE clinical isolates (56 *E. coli*, 60 *E. cloacae*, and 34 *K. pneumoniae*) were collected from the First Affiliated Hospital of Wenzhou Medical University, Wenzhou, Zhejiang, China. Species identification was performed by the matrix-assisted laser desorption ionization time-of-flight mass spectrometry (Bruker Daltonics, US). A total of 75 CZA-susceptible (28 *E. coli*, 30 *E. cloacae*, and 17 *K. pneumoniae*) and 75 CZA-resistant (28 *E. coli*, 30 *E. cloacae*, and 17 *K. pneumoniae*) CRE strains were selected and detected by BMD. *E. coli* ATCC 25922 and *K. pneumoniae* ATCC 700603 were used as the reference control strains. We had investigated the resistant mechanisms of *E. cloacae* previously (Liu et al., 2021).

### Antimicrobial Susceptibility Testing

The assessment of the MICs of CZA was performed in triplicate on Cation-adjusted Mueller–Hinton broth by using the BMD. In accordance with the CLSI guidelines (Prater et al., 2019), the MIC breakpoint for CZA provided for *Enterobacterales* was ≤ 8/4 μg/ml (susceptible breakpoint) and ≥16/4 μg/ml (resistant breakpoint).

### Polymerase Chain Reaction and DNA Sequencing

Genomic DNA of all tested clinical isolates was extracted using the Biospin Bacterial Genomic DNA Extraction Kit (Bioflux, Tokyo, Japan) in accordance with the instructions of the manufacturer. The resistant determinants, including carbapenem genes (*bla*_KPC−2_, *bla*_NDM_, *bla*_IMP_, *bla*_VIM_, and *bla*_OXA−23_, *bla*_OXA−48_), ESBL genes (*bla*_SHV_, *bla*_TEM_, *bla*_CTX−M−1_, *bla*_CTX−M−9_, and *bla*_CTX−M−14_), the outer membrane porin genes (*ompC* and *ompF*), and cephalosporinase gene (*AmpC*), were examined by PCR using specific primers (Table 1). The positive PCR products were subsequently sequenced.

**Table 1:** PCR primers used in this study.

**Genes**	**Sequence (5^**′**^ → 3^**′**^)**	**Annealing temperature (**°**C)**	**Amplicon size (bp)**
*bla*_KPC_	F: GCTACACCTAGCTCCACCTTC	52	1,050
	R: TCAGTGCTCTACAGAAAACC		
*bla*_NDM_	F: GGTTTGGCGATCTGGTTTTC	52	621
	R: CGGAATGGCTCACGATC		
*bla*_IMP_	F: CATGGTTTGGTGGTTCTTGT	50	488
	R: ATAATTTGGCGGACTTTGGC		
*bla*_VIM_	F: GATGGTGTTTGGTCGCATA	58	390
	R: CGAATGCGCAGCACCAG		
*bla*_OXA−23_	F: ACTTGCTATGTGGTTGCTTCTCTT	55	797
	R: TTCAGCTGTTTTAATGATTTCATCA		
*bla*_OXA−48_	F: TTGGTGGCATCGATTATCGG	58	744
	R: GAGCACTTCTTTTGTGATGGC		
*bla*_SHV_	F: AGCCGCTTGAGCAAATTAAAC	60	713
	R: ATCCCGCAGATAAATCACCAC		
*bla*_TEM_	F: CATTTCCGTGTCGCCCTTATTC	60	800
	R: CGTTCATCCATAGTTGCCTGAC		
*bla*_CTX−M−1_	F: AAAAATCACTGCGTCAGTTCAC	55	867
	R: ACAAACCGTTGGTGACGATT		
*bla*_CTX−M−9_	F: TATTGGGAGTTTGAGATGGT	50	933
	R: TCCTTCAACTCAGCAAAAGT		
*bla*_CTX−M−14_	F: CTGCTTAATCAGCCTGTCGA	50	230
	R: TCAGTGCGATCCAGACGAAA		
*ompC*	F: GAGAATGGACTTGCCGACTG	55	1,289
	R: CGAACGGTCGCAAGAGTA		
*ompF*	F: CAGAACTTATTGACGGCAG	55	1,410
	R: CGGGACGTTCATCGGCAC		
*bla*_AmpC_	F: ACTTACTTCAACTCGCGACG	55	663
	R: TAAACACCACATATGTTCCG		

### Screening CZA Resistance in CRE Clinical Isolates Using the SS CZA Medium

**Figure 1:**
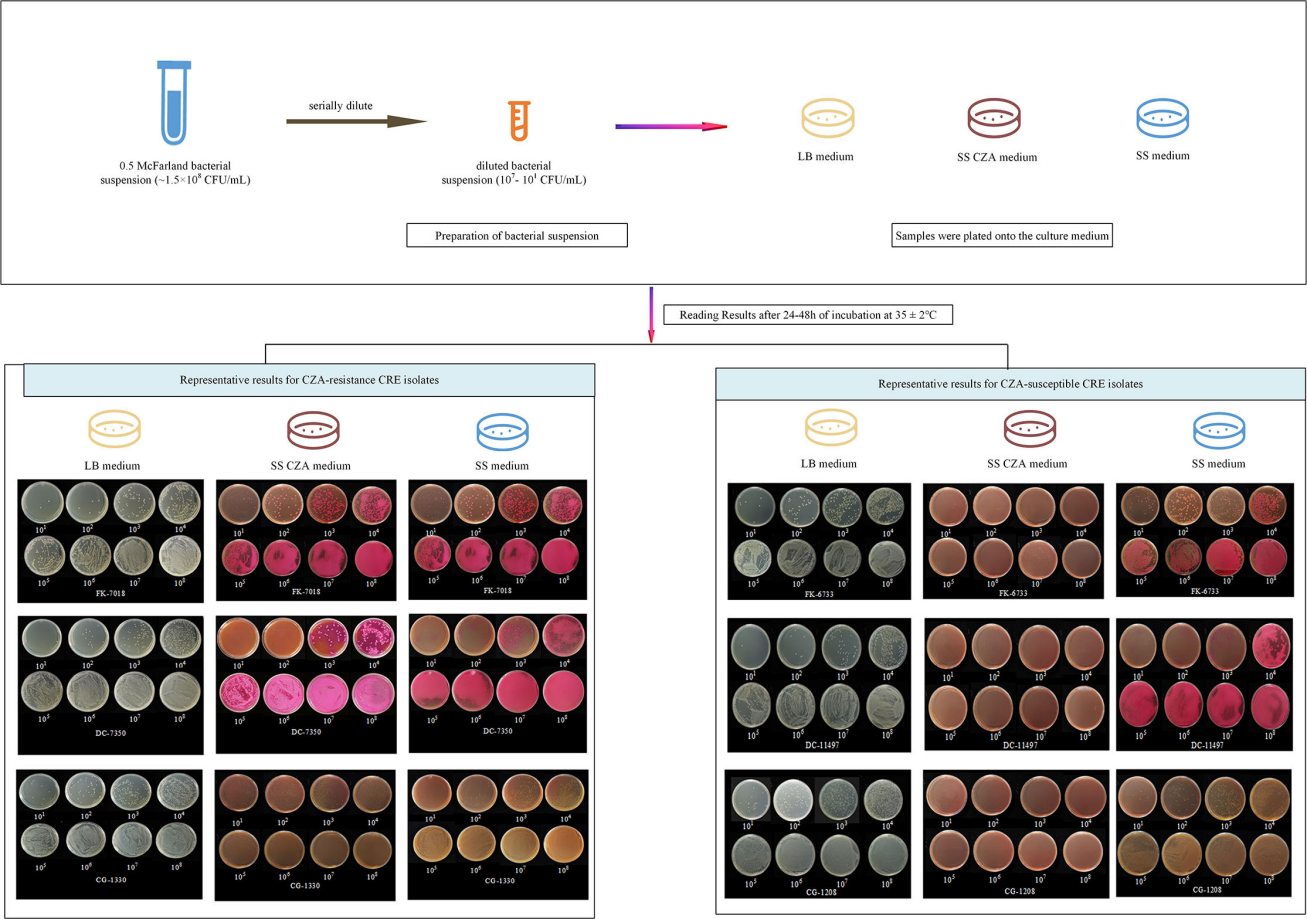
Screening procedure and representative results with the SS CZA medium, SS medium, and LB medium. For the preparation of bacterial suspensions, 0.85% of sterile saline solution was used to adjust the bacterial suspensions to 0.5 McFarland (~1.5 × 10^8^ CFU/ml), followed by serial dilution of the bacterial suspensions with 0.85% sterile saline solution or the prepared stool suspensions to 10^7^, 10^6^, 10^5^, 10^4^, 10^3^, 10^2^, and 10^1^ CFU/ml. Then, 100 μl of each diluted bacterial suspension was plated onto the LB medium, SS CZA medium, and SS medium. All tested plates were incubated overnight at 37°C for 24–48 h, after which the bacterial colonies were counted. We presented two representative results for CZA-resistant and -susceptible CRE isolates. For CZA-resistant CRE isolates (FK-7018, DC-7350, and CG-1330), they could be recovered within 24 h on SS CZA medium, SS medium, and LB medium plates by using an inoculum of 10^1^-10^8^ CFU/ml. In contrast, growth of the CZA-susceptible CRE isolates (FK-6733, DC-11497, and CG-1208) was inhibited within 24 h on SS CZA medium plates, not on SS medium or LB medium plates, when an inoculum of 10^1^-10^8^ CFU/ml.

Sadek et al.'s (2020) method was adopted, albeit with slight modifications, with at least two independent repeated experiments. The specific experimental procedure is illustrated in Figure 1 and described in the following steps.

#### Step 1: Preparation of the Culture Medium

Three different culture media were prepared, including Luria–Bertani (LB) agar medium, SS agar medium, and SS agar medium with CZA (SS CZA medium). These media were prepared according to the instructions using the following formulas: (i) LB agar medium: tryptone 10 g/L, yeast extract 5 g/L, NaCl 10 g/L, agar 15 g/L, distilled water; (ii) SS agar medium: SS agar 62 g/L, distilled water; (iii) SS CZA medium: SS agar 62 g/L, distilled water, CAZ 6 mg/L, AVI 4 mg/L. Specifically, the LB and SS agar media were autoclaved at 121 and 105°C for 20 min, respectively, to dissolve them. After the SS CZA medium was cooled at 50°C for 1 h, the antibiotic stock solutions of CAZ (final concentration of 6 μg/ml) and AVI (final concentration of 4 μg/ml) were added to it. These configured media were then poured into empty plates and stored at 4°C until further use.

#### Step 2: Preparation of Bacterial Suspensions and Clinical Simulated Stool Samples

For the preparation of bacterial suspensions, 0.85% sterile saline solution was used to adjust the bacterial suspensions to 0.5 McFarland (~1.5 × 10^8^ CFU/ml), followed by serial dilution of the bacterial suspensions with 0.85% sterile saline solution to 10^7^, 10^6^, 10^5^, 10^4^, 10^3^, 10^2^, and 10^1^ CFU/ml. The bacterial suspensions were used within 1.5 h of preparation. Then, 0.85% sterile saline solution with or without reference control strains (ATCC 25922 or ATCC 700603) was used as negative controls.

For the preparation of clinical simulated stool samples, fresh pooled feces from healthy volunteers were collected and prepared into suspensions (concentration of 10 g/100 ml) with 0.85% sterile saline solution. Meanwhile, 0.85% sterile saline solution was used to adjust the bacterial suspensions to 0.5 McFarland (~1.5 × 10^8^ CFU/ml). Then, the clinically simulated stool samples were prepared and the bacterial suspensions were serially diluted with the prepared stool suspensions to 10^7^, 10^6^, 10^5^, 10^4^, 10^3^, 10^2^, and 10^1^ CFU/ml. The clinical simulated stool samples were to be used within 1.5 h of preparation. The stool suspensions with or without reference control strains (*E. coli* ATCC 25922 and *K. pneumoniae* ATCC 700603) were used as negative controls.

#### Step 3: Samples Were Plated Onto the Culture Medium

In this step, 100 μl of each diluted bacterial suspension was plated onto the SS agar plates and SS CZA plates. Meanwhile, to quantify the viable bacterial count in each dilution step, LB agar plates were inoculated with the same amount of the abovementioned bacterial suspension. The screening procedure for clinically simulated fecal samples was the same as that for pure strains. To eliminate false-positive results contributed by fecal samples, 100 μl of 0.85% sterile saline solution or fecal suspensions without the tested strains were plated onto these three media. All tested plates were incubated overnight at 37°C for 24 h, and the bacterial colonies were enumerated. If no bacterial growth was detected within 24 h, the plates were continuously cultured for 48 h to assess the negativity of the culture. The lowest limit of detection for the studied strains was determined by the SS CAZ medium.

#### Step 4: Result Interpretations and Analysis

In this study, the sensitivity and specificity cutoff values for the detection of CZA-resistant CRE isolates were set to 1 × 10^3^ CFU/ml (Nordmann et al., 2012). In other words, the results were considered positive when CZA-resistant CRE strains could grow on the SS CZA medium at ≤ 1 × 10^3^ CFU/ml, while the CZA-susceptible CRE strains that showed a visible growth with an inoculum of >1 × 10^3^ CFU/ml were considered as negative. In order to exclude the false-positive/-negative culture results, the results were considered interpretable under the following conditions: (i) the LB, SS, and SS CZA agar plates with 100 μl of 0.85% sterile saline solution without tested strains showed no bacterial growth; (ii) bacterial growth was visible on the LB agar plate inoculated with 100 μl of the fecal suspensions without tested strains, and no growth was detected on the SS and SS CZA agar plates; and (iii) bacterial growth was observed on both the SS and LB agar plates inoculated with either the pure bacterial suspension or simulated specimen suspension, and the bacterial colonies on these media were enumerated to ensure the accuracy of the McFarland standard or the bacterial load and reliability of our findings.

## Results

### The Tested CRE Clinical Strains Demonstrated Different Resistance Determinants

A total of 150 non-duplicate CRE clinical isolates were obtained, which included 75 CZA-resistant and 75 CZA-susceptible isolates, to evaluate the performances of the SS CZA medium. Their MIC of CZA ranged from ≤ 0.125 to ≥256 μg/ml (Table 2). The PCR results demonstrated that NDM was the main resistance determinant in CZA-resistant *E. coli* and *E. cloacae*. We also detected NDM in a CZA-susceptible *E. cloacae* strain (CG-1479). In addition, the resistance determinants of CZA-resistant *E. cloacae* CG-996 and CG-1045 were *bla*_OXA−23_ and IMP, respectively. KPC-2 was detected in *K. pneumoniae*. KPC-33, a KPC-2 variant with the D179Y mutation in the omega loop, was detected in FK-8696. A high expression of AmpC, impermeability, or efflux pump was detected in CG1212, CG1249, and other strains (Liu et al., 2021). In addition, carbapenemase was found to coexist with other ESBL genes, such as *bla*_TEM−1_, *bla*_CTX−M−1_, *bla*_CTX−M−9_, *bla*_CTX−M−14_, *bla*_SHV_, and *bla*_TEM−1_, as well as the outer membrane porin-encoding genes *ompC* and *ompF* in several strains (Table 2).

**Table 2:** Summary of MIC values of the CZA for the tested isolates and the lowest detection limits of the SS CZA medium, SS medium, and LB medium.

**Isolates**	**Species**	**MIC of CZA (μg/ml)**	**CZA Resistant Phenotype**	**Resistance determinant(s)**	**Lowest detection limit (CFU/ml) of**					
					**SS CZA medium** ^**a**^		**SS medium**		**LB medium**	
					**Culture**	**Stools**	**Culture**	**Stools**	**Culture**	**Stools**
DC-2003	*E. coli*	64	R	CTX-M-1, CTX-M-9, OmpF	10^1^	10^3^	10^1^	10^1^	10^1^	10^1^
DC-5128	*E. coli*	>256	R	NDM, CTX-M-1, CTX-M-9, OmpC, OmpF	10^1^	10^1^	10^1^	10^1^	10^1^	10^1^
DC-7114	*E. coli*	>256	R	NDM, TEM, CTX-M-1, OmpC	10^1^	10^1^	10^1^	10^1^	10^1^	10^1^
DC-7143	*E. coli*	>256	R	NDM, TEM, OmpC	10^1^	10^1^	10^1^	10^1^	10^1^	10^1^
DC-7333	*E. coli*	>256	R	NDM, TEM, CTX-M-1, CTX-M-9, OmpC	10^1^	10^1^	10^1^	10^1^	10^1^	10^1^
DC-7350	*E. coli*	>256	R	NDM, TEM, CTX-M-1	10^1^	10^1^	10^1^	10^1^	10^1^	10^1^
DC-7368	*E. coli*	>256	R	NDM, TEM, CTX-M-1	10^2^	10^1^	10^1^	10^1^	10^1^	10^1^
DC-7523	*E. coli*	>256	R	NDM, CTX-M-1, CTX-M-9, OmpC	10^1^	10^1^	10^1^	10^1^	10^1^	10^1^
DC-7658	*E. coli*	>256	R	NDM, TEM, CTX-M-1, CTX-M-9	10^1^	10^1^	10^1^	10^1^	10^1^	10^1^
DC-7706	*E. coli*	>256	R	NDM, TEM, CTX-M-1, CTX-M-9, OmpF	10^1^	10^1^	10^1^	10^1^	10^1^	10^1^
DC-7741	*E. coli*	>256	R	NDM, TEM, CTX-M-1, OmpC	10^1^	10^1^	10^1^	10^1^	10^1^	10^1^
DC-7781	*E. coli*	>256	R	NDM, TEM, CTX-M-1, OmpC	10^2^	10^1^	10^1^	10^1^	10^1^	10^1^
DC-7782	*E. coli*	>256	R	NDM, TEM, CTX-M-1, OmpC	10^1^	10^1^	10^1^	10^1^	10^1^	10^1^
DC-7911	*E. coli*	>256	R	NDM, CTX-M-9, OmpF	10^1^	10^1^	10^1^	10^1^	10^1^	10^1^
DC-7914	*E. coli*	>256	R	NDM, TEM, CTX-M-9, OmpF	10^1^	10^2^	10^1^	10^1^	10^1^	10^1^
DC-7956	*E. coli*	>256	R	NDM, TEM, CTX-M-9, OmpF	10^1^	10^2^	10^1^	10^1^	10^1^	10^1^
DC-8111	*E. coli*	>256	R	NDM, TEM, CTX-M-1, CTX-M-9, OmpC	10^1^	10^2^	10^1^	10^1^	10^1^	10^1^
DC-8439	*E. coli*	>256	R	NDM	10^2^	10^1^	10^1^	10^1^	10^1^	10^1^
DC-8466	*E. coli*	>256	R	NDM	10^1^	10^1^	10^1^	10^1^	10^1^	10^1^
DC-8647	*E. coli*	>256	R	NDM	10^1^	10^1^	10^1^	10^1^	10^1^	10^1^
DC-8823	*E. coli*	>256	R	NDM	10^1^	10^1^	10^1^	10^1^	10^1^	10^1^
DC-8855	*E. coli*	>256	R	NDM	10^1^	10^1^	10^1^	10^1^	10^1^	10^1^
DC-8896	*E. coli*	>256	R	NDM	10^1^	10^1^	10^1^	10^1^	10^1^	10^1^
DC-10494	*E. coli*	>256	R	NDM	10^2^	10^3^	10^1^	10^1^	10^1^	10^1^
DC-10527	*E. coli*	>256	R	NDM	10^1^	10^1^	10^1^	10^1^	10^1^	10^1^
DC-10921	*E. coli*	>256	R	NDM	10^2^	10^3^	10^1^	10^1^	10^1^	10^1^
DC-11403	*E. coli*	>256	R	NDM	10^1^	10^1^	10^1^	10^1^	10^1^	10^1^
DC-11552	*E. coli*	>256	R	NDM	10^1^	10^1^	10^1^	10^1^	10^1^	10^1^
ATCC 25922	*E. coli*	0.125	S	Reference	>10^8^	>10^8^	10^1^	10^1^	10^1^	10^1^
DC-1918	*E. coli*	4	S	CTX-M-9, OmpF	>10^8^	>10^8^	10^1^	10^1^	10^1^	10^1^
DC-3285	*E. coli*	0.5	S	KPC-2, CTX-M-1	>10^8^	>10^8^	10^1^	10^1^	10^1^	10^1^
DC-5113	*E. coli*	1	S	KPC-2, TEM, CTX-M-1, CTX-M-9	>10^8^	>10^8^	10^1^	10^1^	10^1^	10^1^
DC-5293	*E. coli*	0.125	S	TEM, SHV	>10^8^	>10^8^	10^1^	10^1^	10^1^	10^1^
DC-6669	*E. coli*	1	S	TEM, CTX-M-9, OmpF	>10^8^	>10^8^	10^1^	10^1^	10^1^	10^1^
DC-6834	*E. coli*	1	S	CTX-M-1, OmpF	>10^8^	>10^8^	10^1^	10^1^	10^1^	10^1^
DC-6856	*E. coli*	0.25	S	KPC-2, CTX-M-1, CTX-M-9, OmpF	>10^8^	>10^8^	10^1^	10^1^	10^1^	10^1^
DC-6896	*E. coli*	≤ 0.125	S	KPC-2, CTX-M-1, CTX-M-9, OmpF	>10^8^	>10^8^	10^1^	10^1^	10^1^	10^1^
DC-8535	*E. coli*	4	S	CTX-M-9, SHV	>10^8^	>10^8^	10^1^	10^1^	10^1^	10^1^
DC-8873	*E. coli*	1	S	TEM, SHV	>10^8^	>10^8^	10^1^	10^1^	10^1^	10^1^
DC-10694	*E. coli*	0.5	S	CTX-M-1, CTX-M-9, TEM, SHV	>10^8^	>10^8^	10^1^	10^1^	10^1^	10^1^
DC-10709	*E. coli*	0.25	S	CTXM-1, TEM, SHV	>10^8^	>10^8^	10^1^	10^1^	10^1^	10^1^
DC-10740	*E. coli*	0.5	S	CTX-M-9, SHV	>10^8^	>10^8^	10^1^	10^1^	10^1^	10^1^
DC-11104	*E. coli*	0.25	S	CTX-M-1, TEM, SHV	>10^8^	>10^8^	10^1^	10^1^	10^1^	10^1^
DC-11305	*E. coli*	0.5	S	CTX-M-1, TEM, SHV	>10^8^	>10^8^	10^1^	10^1^	10^1^	10^1^
DC-11308	*E. coli*	0.5	S	CTX-M-1, TEM, SHV	>10^8^	>10^8^	10^1^	10^1^	10^1^	10^1^
DC-11497	*E. coli*	4	S	CTX-M-1	>10^8^	>10^8^	10^1^	10^1^	10^1^	10^1^
DC-11537	*E. coli*	0.5	S	CTX-M-1, TEM, SHV	>10^8^	>10^8^	10^1^	10^1^	10^1^	10^1^
DC-11581	*E. coli*	0.5	S	KPC-2	>10^8^	>10^8^	10^1^	10^1^	10^1^	10^1^
DC-11720	*E. coli*	2	S	KPC-2	>10^8^	>10^8^	10^1^	10^1^	10^1^	10^1^
DC-12735	*E. coli*	2	S	KPC-2	>10^8^	>10^8^	10^1^	10^1^	10^1^	10^1^
DC-13016	*E. coli*	1	S	CTX-M-9, SHV	>10^8^	>10^8^	10^1^	10^1^	10^1^	10^1^
DC-13149	*E. coli*	2	S	CTX-M-1, CTX-M-9	>10^8^	>10^8^	10^1^	10^1^	10^1^	10^1^
DC-13346	*E. coli*	4	S	CTX-M-1, TEM, SHV	>10^8^	>10^8^	10^1^	10^1^	10^1^	10^1^
DC-14122	*E. coli*	0.125	S	CTX-M-1	>10^8^	>10^8^	10^1^	10^1^	10^1^	10^1^
DC-14323	*E. coli*	0.5	S	CTX-M-1, CTX-M-9, TEM, SHV	>10^8^	>10^8^	10^1^	10^1^	10^1^	10^1^
DC-14324	*E. coli*	2	S	KPC-2	>10^8^	>10^8^	10^1^	10^1^	10^1^	10^1^
DC-14351	*E. coli*	1	S	CTXM-9, SHV	>10^8^	>10^8^	10^1^	10^1^	10^1^	10^1^
CG-175^b^ (Liu et al., 2021)	*E. cloacae*	256	R	NDM, CTX-M-9, CTX-M-14	10^1^	10^1^	10^1^	10^1^	10^1^	10^1^
CG-586	*E. cloacae*	>256	R	NDM, CTX-M-1	10^1^	10^1^	10^1^	10^1^	10^1^	10^1^
CG-662	*E. cloacae*	>256	R	NDM	10^1^	10^1^	10^1^	10^1^	10^1^	10^1^
CG-698	*E. cloacae*	256	R	NDM	10^1^	10^1^	10^1^	10^1^	10^1^	10^1^
CG-749	*E. cloacae*	>256	R	NDM, efflux pump	10^1^	10^1^	10^1^	10^1^	10^1^	10^1^
CG-838	*E. cloacae*	64	R	NDM	10^1^	10^2^	10^1^	10^1^	10^1^	10^1^
CG-901	*E. cloacae*	>256	R	NDM	10^1^	10^1^	10^1^	10^1^	10^1^	10^1^
CG-983	*E. cloacae*	>256	R	NDM, efflux pump	10^1^	10^1^	10^1^	10^1^	10^1^	10^1^
CG-996	*E. cloacae*	128	R	OXA-23, TEM	10^1^	10^1^	10^1^	10^1^	10^1^	10^1^
CG-1045	*E. cloacae*	>256	R	IMP	10^1^	10^1^	10^1^	10^1^	10^1^	10^1^
CG-1075	*E. cloacae*	>256	R	NDM, efflux pump	10^1^	10^1^	10^1^	10^1^	10^1^	10^1^
CG-1090	*E. cloacae*	>256	R	NDM	10^1^	10^1^	10^1^	10^1^	10^1^	10^1^
CG-1608	*E. cloacae*	>256	R	NDM, SHV, efflux pump	10^1^	10^1^	10^1^	10^1^	10^1^	10^1^
CG-1819	*E. cloacae*	128	R	NDM, CTX-M-9	10^1^	10^1^	10^1^	10^1^	10^1^	10^1^
CG-1141	*E. cloacae*	>256	R	TEM, SHV	10^1^	10^1^	10^1^	10^1^	10^1^	10^1^
CG-1212	*E. cloacae*	>256	R	TEM, CTXM-14, impermeability, efflux pump	10^1^	10^2^	10^1^	10^1^	10^1^	10^1^
CG-1249	*E. cloacae*	64	R	AmpC, impermeability	10^1^	10^2^	10^1^	10^1^	10^1^	10^1^
CG-1257	*E. cloacae*	>256	R	IMP	10^1^	10^1^	10^1^	10^1^	10^1^	10^1^
CG-1280	*E. cloacae*	>256	R	impermeability	10^1^	10^2^	10^1^	10^1^	10^1^	10^1^
CG-1330	*E. cloacae*	>256	R	NDM	10^1^	10^1^	10^1^	10^1^	10^1^	10^1^
CG-1381	*E. cloacae*	>256	R	OXA-23	10^1^	10^1^	10^1^	10^1^	10^1^	10^1^
CG-1498	*E. cloacae*	>256	R	IMP	10^1^	10^1^	10^1^	10^1^	10^1^	10^1^
CG-1574	*E. cloacae*	>256	R	NDM, CTXM-14	10^1^	10^1^	10^1^	10^1^	10^1^	10^1^
CG-1591	*E. cloacae*	>256	R	IMP	10^1^	10^1^	10^1^	10^1^	10^1^	10^1^
CG-1593	*E. cloacae*	>256	R	IMP	10^1^	10^1^	10^1^	10^1^	10^1^	10^1^
CG-1737	*E. cloacae*	>256	R	NDM	10^2^	10^3^	10^1^	10^1^	10^1^	10^1^
CG-1778	*E. cloacae*	>256	R	NDM, efflux pump	10^1^	10^1^	10^1^	10^1^	10^1^	10^1^
CG-1779	*E. cloacae*	>256	R	NDM	10^1^	10^1^	10^1^	10^1^	10^1^	10^1^
CG-1781	*E. cloacae*	>256	R	NDM	10^1^	10^1^	10^1^	10^1^	10^1^	10^1^
CG-1813	*E. cloacae*	>256	R	IMP, efflux pump	10^1^	10^1^	10^1^	10^1^	10^1^	10^1^
CG-648	*E. cloacae*	≤ 0.125	S	KPC-2, AmpC	>10^8^	>10^8^	10^1^	10^1^	10^1^	10^1^
CG-701	*E. cloacae*	0.5	S	SHV, TEM, CTX-M-1, AmpC	>10^8^	>10^8^	10^1^	10^1^	10^1^	10^1^
CG-737	*E. cloacae*	0.5	S	AmpC	>10^8^	>10^8^	10^1^	10^1^	10^1^	10^1^
CG-741	*E. cloacae*	0.5	S	AmpC	>10^8^	>10^8^	10^1^	10^1^	10^1^	10^1^
CG-864	*E. cloacae*	1	S	AmpC	>10^8^	>10^8^	10^1^	10^1^	10^1^	10^1^
CG-934	*E. cloacae*	1	S	AmpC	>10^8^	>10^8^	10^1^	10^1^	10^1^	10^1^
CG-1038	*E. cloacae*	0.5	S	KPC-2, AmpC	>10^8^	>10^8^	10^1^	10^1^	10^1^	10^1^
CG-1048	*E. cloacae*	1	S	CTX-M-14, AmpC	>10^8^	>10^8^	10^1^	10^1^	10^1^	10^1^
CG-1050	*E. cloacae*	2	S	SHV, AmpC	>10^8^	>10^8^	10^1^	10^1^	10^1^	10^1^
CG-1051	*E. cloacae*	2	S	SHV, TEM, CTX-M-1, AmpC	>10^8^	>10^8^	10^1^	10^1^	10^1^	10^1^
CG-1479	*E. cloacae*	1	S	NDM, SHV, CTX-M-9, CTX-M-14, AmpC	>10^8^	>10^8^	10^1^	10^1^	10^1^	10^1^
CG-1506	*E. cloacae*	0.5	S	SHV, CTX-M-14	>10^8^	>10^8^	10^1^	10^1^	10^1^	10^1^
CG-1081	*E. cloacae*	4	S	TEM, AmpC, efflux pump	>10^8^	>10^8^	10^1^	10^1^	10^1^	10^1^
CG-1144	*E. cloacae*	0.25	S	impermeability	>10^8^	>10^8^	10^1^	10^1^	10^1^	10^1^
CG-1159	*E. cloacae*	2	S	AmpC, impermeability	>10^8^	>10^8^	10^1^	10^1^	10^1^	10^1^
CG-1181	*E. cloacae*	4	S	SHV, TEM, CTX-M-1, CTX-M-9, CTX-M-14, impermeability	>10^8^	>10^8^	10^1^	10^1^	10^1^	10^1^
CG-1208	*E. cloacae*	8	S	TEM, CTXM-9, CTXM-14, AmpC, impermeability	>10^8^	>10^8^	10^1^	10^1^	10^1^	10^1^
CG-1231	*E. cloacae*	1	S	AmpC, impermeability	>10^8^	>10^8^	10^1^	10^1^	10^1^	10^1^
CG-1236	*E. cloacae*	2	S	TEM, AmpC, impermeability	>10^8^	>10^8^	10^1^	10^1^	10^1^	10^1^
CG-1250	*E. cloacae*	2	S	AmpC, impermeability	>10^8^	>10^8^	10^1^	10^1^	10^1^	10^1^
CG-1252	*E. cloacae*	1	S	impermeability	>10^8^	>10^8^	10^1^	10^1^	10^1^	10^1^
CG-1281	*E. cloacae*	1	S	impermeability	>10^8^	>10^8^	10^1^	10^1^	10^1^	10^1^
CG-1457	*E. cloacae*	8	S	AmpC	>10^8^	>10^8^	10^1^	10^1^	10^1^	10^1^
CG-1461	*E. cloacae*	4	S	AmpC, impermeability	>10^8^	>10^8^	10^1^	10^1^	10^1^	10^1^
CG-1522	*E. cloacae*	4	S	TEM, CTX-M-1, AmpC, impermeability, efflux pump	>10^8^	>10^8^	10^1^	10^1^	10^1^	10^1^
CG-1532	*E. cloacae*	1	S	Impermeability, efflux pump	>10^8^	>10^8^	10^1^	10^1^	10^1^	10^1^
CG-1547	*E. cloacae*	2	S	KPC-2, SHV, TEX-M-9, CTXM-14, efflux pump	>10^8^	>10^8^	10^1^	10^1^	10^1^	10^1^
CG-1563	*E. cloacae*	4	S	SHV, TEM, CTX-M-14, impermeability	>10^8^	>10^8^	10^1^	10^1^	10^1^	10^1^
CG-1565	*E. cloacae*	4	S	SHV, AmpC, impermeability, efflux pump	>10^8^	>10^8^	10^1^	10^1^	10^1^	10^1^
CG-1581	*E. cloacae*	2	S	TEM, AmpC, impermeability, efflux pump	>10^8^	>10^8^	10^1^	10^1^	10^1^	10^1^
FK-2784	*K. pneumoniae*	64	R	KPC-2	10^1^	10^1^	10^1^	10^1^	10^1^	10^1^
FK-3246	*K. pneumoniae*	>256	R	KPC-2	10^1^	10^1^	10^1^	10^1^	10^1^	10^1^
FK-4722	*K. pneumoniae*	>256	R	KPC-2	10^1^	10^1^	10^1^	10^1^	10^1^	10^1^
FK-7018	*K. pneumoniae*	>256	R	NDM	10^1^	10^1^	10^1^	10^1^	10^1^	10^1^
FK-7079	*K. pneumoniae*	>256	R	CTX-M-9, SHV, TEM	10^1^	10^1^	10^1^	10^1^	10^1^	10^1^
FK-7173	*K. pneumoniae*	>256	R	NDM	10^1^	10^1^	10^1^	10^1^	10^1^	10^1^
FK-7513	*K. pneumoniae*	>256	R	NDM	10^1^	10^1^	10^1^	10^1^	10^1^	10^1^
FK-7710	*K. pneumoniae*	>256	R	NDM	10^1^	10^1^	10^1^	10^1^	10^1^	10^1^
FK-7786	*K. pneumoniae*	64	R	NDM	10^1^	10^1^	10^1^	10^1^	10^1^	10^1^
FK-7921	*K. pneumoniae*	>256	R	NDM	10^1^	10^1^	10^1^	10^1^	10^1^	10^1^
FK-7978	*K. pneumoniae*	>256	R	NDM	10^1^	10^1^	10^1^	10^1^	10^1^	10^1^
FK-8401	*K. pneumoniae*	>256	R	NDM	10^1^	10^1^	10^1^	10^1^	10^1^	10^1^
FK-8696	*K. pneumoniae*	>256	R	KPC-33, CTX-M-9, SHV, TEM	10^1^	10^1^	10^1^	10^1^	10^1^	10^1^
FK-8966	*K. pneumoniae*	>256	R	NDM	10^1^	10^1^	10^1^	10^1^	10^1^	10^1^
FK-9102	*K. pneumoniae*	>256	R	NDM	10^1^	10^1^	10^1^	10^1^	10^1^	10^1^
FK-9250	*K. pneumoniae*	>256	R	NDM	10^1^	10^1^	10^1^	10^1^	10^1^	10^1^
FK-9283	*K. pneumoniae*	>256	R	NDM	10^1^	10^1^	10^1^	10^1^	10^1^	10^1^
ATCC 700603	*K. pneumoniae*	≤ 0.125	S	Reference	>10^8^	>10^8^	10^1^	10^1^	10^1^	10^1^
FK-2731	*K. pneumoniae*	0.5	S	KPC-2	>10^8^	>10^8^	10^1^	10^1^	10^1^	10^1^
FK-2742	*K. pneumoniae*	0.5	S	KPC-2	>10^8^	>10^8^	10^1^	10^1^	10^1^	10^1^
FK-2836	*K. pneumoniae*	4	S	KPC-2, IMP	>10^8^	>10^8^	10^1^	10^1^	10^1^	10^1^
FK-2877	*K. pneumoniae*	2	S	KPC-2	>10^8^	>10^8^	10^1^	10^1^	10^1^	10^1^
FK-2970	*K. pneumoniae*	1	S	KPC-2	>10^8^	>10^8^	10^1^	10^1^	10^1^	10^1^
FK-3006	*K. pneumoniae*	1	S	KPC-2, IMP	>10^8^	>10^8^	10^1^	10^1^	10^1^	10^1^
FK-3020	*K. pneumoniae*	1	S	IMP	>10^8^	>10^8^	10^1^	10^1^	10^1^	10^1^
FK-3093	*K. pneumoniae*	4	S	KPC-2	>10^8^	>10^8^	10^1^	10^1^	10^1^	10^1^
FK-3142	*K. pneumoniae*	2	S	KPC-2	>10^8^	>10^8^	10^1^	10^1^	10^1^	10^1^
FK-6699	*K. pneumoniae*	0.5	S	KPC-2	>10^8^	>10^8^	10^1^	10^1^	10^1^	10^1^
FK-6701	*K. pneumoniae*	1	S	KPC-2	>10^8^	>10^8^	10^1^	10^1^	10^1^	10^1^
FK-6703	*K. pneumoniae*	1	S	KPC-2	>10^8^	>10^8^	10^1^	10^1^	10^1^	10^1^
FK-6719	*K. pneumoniae*	2	S	KPC-2	>10^8^	>10^8^	10^1^	10^1^	10^1^	10^1^
FK-6723	*K. pneumoniae*	2	S	KPC-2	>10^8^	>10^8^	10^1^	10^1^	10^1^	10^1^
FK-6728	*K. pneumoniae*	2	S	KPC-2	>10^8^	>10^8^	10^1^	10^1^	10^1^	10^1^
FK-6733	*K. pneumoniae*	8	S	KPC-2	>10^8^	>10^8^	10^1^	10^1^	10^1^	10^1^
FK-6735	*K. pneumoniae*	4	S	KPC-2	>10^8^	>10^8^	10^1^	10^1^	10^1^	10^1^

*E. coli, Escherichia coli; E. cloacae, Enterobacter cloacae; K. pneumoniae, Klebsiella pneumoniae; MIC, minimal inhibitory concentration; CZA, ceftazidime-avibactam; R, resistant; S, susceptible; NDM, New Delhi Metallo-beta-lactamase; KPC, K. pneumoniae carbapenemase; TEM,Temoneira β-lactamase; CTX-M, cefotaximase-Munich-type β-lactamase; SHV, sulfhydryl reagent variable β-lactamase; OXA, oxacillinase; AmpC, AmpC-type β-lactamase; IMP, IMP-type metallo-β-lactamase; OmpC/OmpF, outer membrane porins genes*.

^a^*means that cutoff values were set at 1 × 10^3^ CFU/mL, i.e., the results were considered as positive when CZA-resistant CRE strains can grow on SS CZA medium at ≤ 1 × 10^3^ CFU/mL, while the CZA-susceptible CRE strains have a visible growth using an inoculum of >1 × 10^3^ CFU/mL were considered as negative*.

^b^*means that the resistant mechanisms of E. cloacae were investigated in our published study*.

### SS CZA Medium Demonstrated a Great Ability for Screening CZA Resistance Among the CRE Isolates

The results of the SS CZA medium toward the detection of CZA-resistant and CZA-susceptible CRE isolates are shown in Table 2 and Figure 1. All CZA-resistant and -susceptible CRE isolates could be recovered within 24 h on SS medium and LB medium plates by using an inoculum of 10^1^-10^8^ CFU/ml. For CZA-resistant CRE isolates, they could be recovered within 24 h on SS CZA medium even at the low dilution gradient of 10^1^ to 10^2^ CFU/ml (below the cutoff value). In contrast, growth of the CZA-susceptible CRE isolates was inhibited within 24–48 h on SS CZA medium even at the highest dilution gradient of 10^8^ CFU/ml (above the cutoff value). Furthermore, we assessed the ability of the SS CZA medium to screen the CZA-resistant CRE isolates from clinically simulated specimens. As expected, the clinically simulated stool samples with CZA-resistant CRE isolates could grow on the SS CZA medium within 24 h using an inoculum of 10^1^ to 10^3^ CFU/ml (not greater than the cutoff value). In contrast, the clinically simulated stool samples with CZA-susceptible CRE isolates did not grow within 24–48 h even with an inoculum size of 10^8^ CFU/ml (above the cutoff value). With the same cutoff value (setting at 1 × 10^3^ CFU/ml), the lower detection limit for pure CZA-resistant CRE strains and clinically simulated stool samples with CZA-resistant CRE isolates ranged from 10^1^ to 10^2^ and 10^1^ to 10^3^ CFU/ml, respectively. In contrast, the lower detection limit for the pure CZA-susceptible isolates and clinically simulated stool samples with the CZA-susceptible isolates was >10^8^ CFU/ml. These data indicate that the sensitivity and specificity of the SS CZA-selective medium for detecting CZA-resistant CRE isolates was 100% (using the same cutoff value, setting at 1 × 10^3^ CFU/ml), both in pure clinical strain specimens and clinically simulated stool samples with CZA-resistant CRE isolates.

### SS CZA Medium Had Great Storage Stability

In addition, the storage stability of the SS CZA medium was evaluated as per the method of Sadek et al. The reference strains (*Staphylococcus aureus* ATCC 25923, *Enterococcus faecalis* ATCC 29212, *E. coli* ATCC 25922, and *K. pneumoniae* ATCC 700603) were selected and subcultured every day onto the SS CZA medium from a single batch of medium stored at 4°C. No visible bacterial growth was observed for at least a 7-day period.

## Discussion

We designed an SS CZA medium based on the work of Sadek et al., albeit with some modifications (Sadek et al., 2020). Specifically, the SS agar medium is a strong selective medium containing sodium citrate and bile salts to inhibit the growth of *Candida* and Gram-positive bacteria, such as *Staphylococcus* and *Enterococcus* strains. Unlike the SuperCAZ/AVI medium, certain antibiotics that inhibit the growth of fungi and Gram-positive bacteria, such as amphotericin B and daptomycin, are not required to be added specifically to the SS CZA medium. Therefore, when compared with the SuperCAZ/AVI medium, our SS CZA medium eliminates several operational steps and saves on the incurred costs (only CZA needs to be added into the SS agar medium).

Next, we tested the performance of the SS CZA medium for screening CZA resistance among CRE. Consistent with the results of Sadek et al. (2020), we found that the lower detection limits for CZA-resistant CRE isolates were 10^1^ to 10^3^ CFU/ml. Moreover, all CZA-susceptible strains could grow on the LB and SS agar medium at the inoculum size of 10^1^ to 10^8^ CFU/ml, but not on the SS CZA medium plates, including strains showing an MIC close to that of the resistant breakpoint (such as FK-6733, DC-11497, and CG-1208 with an MIC at 8, 4, and 8 μg/ml, respectively), despite extending the incubation period to 48 h. However, different from the report of Sadek et al., which showed that the lower detection limits for CZA-susceptible *E. coli* and *K. pneumoniae* were 10^7^ to >10^8^ CFU/ml and 10^5^ to >10^8^ CFU/ml, respectively, our study showed the lower detection limits for all CZA-susceptible isolates (including *E. coli* and *K. pneumoniae*) as both >10^8^ CFU/ml. Given that the bacterial load in different clinical specimens is not consistent, a low detection limit of the SS CZA screening medium is more conducive to successful clinical application. Cumulatively, these data indicate that the SS CZA medium developed in this study contributes to an effective screening of CZA-resistant strains directly from clinical specimens and has significant clinical application value with the potential for development from the commercial perspective.

Most screening methods for CRE are performed with rectal swab specimens, which is a polymicrobial specimen. We, therefore, tested spiked stools with the same representative collection of CZA-resistant and CZA-susceptible CRE isolates using our SS CZA medium. As expected, the screening effect for simulated clinical specimens was similar to that of pure clinical isolates; the lower detection limits for stools containing the CZA-susceptible isolates and CZA-resistant isolates were >10^8^ CFU/ml and 10^1^ to 10^3^ CFU/ml. These results implicate that the SS CZA medium is a promising selective medium for rapid and direct screening of clinical specimens suspected to contain CZA-resistant strains.

However, there are some limitations to our study. For instance, the SS CZA medium cannot give the exact MIC value, although it can help assign the susceptibility and resistance category quite rapidly, which is closely related to clinical medicine. In addition, we did not assess the screening effect of the SS CZA medium on *P. aeruginosa* or other Gram-negative bacteria, which should be explored in future studies.

## Conclusion

The proposed SS CZA medium exhibited a significant performance for screening CZA-resistant CRE isolates with 100% sensitivity and specificity. Its screening performance was unaffected by the difference in the resistance determinants. In fact, the bacterial load of different clinical specimens was not consistent, and the low detection limit of the SS screening medium was found to be more conducive to its successful clinical application.

## Data Availability Statement

The original contributions presented in the study are included in the article/supplementary material, further inquiries can be directed to the corresponding author/s.

## Author Contributions

WZ conducted the experiments, analyzed the data, and wrote the manuscript. WL, YZ, and LW participated in experiments. HS and HJ took part in the analysis of results. TC and YZ participated in the analysis of results. TZ and QW helped design the study. All authors contributed to the article and approved the submitted version.

## Conflict of Interest

The authors declare that the research was conducted in the absence of any commercial or financial relationships that could be construed as a potential conflict of interest.

## Publisher's Note

All claims expressed in this article are solely those of the authors and do not necessarily represent those of their affiliated organizations, or those of the publisher, the editors and the reviewers. Any product that may be evaluated in this article, or claim that may be made by its manufacturer, is not guaranteed or endorsed by the publisher.
